# History of the Development of Knowledge about the Neuroendocrine Control of Ovulation—Recent Knowledge on the Molecular Background

**DOI:** 10.3390/ijms25126531

**Published:** 2024-06-13

**Authors:** Flóra Szabó, Katalin Köves, Levente Gál

**Affiliations:** 1 Division of Gastroenterology and Nutrition, Children’s Hospital of Richmond, Virginia Commonwealth University, Richmond, VA 23298, USA; flora.szabo@vcuhealth.org; 2 Department of Anatomy, Histology and Embryology, Faculty of Medicine, Semmelweis University, 1094 Budapest, Hungary; 3 Department of Biological Sciences, Virginia Tech, Blacksburg, VA 24061, USA; leventegal@vt.edu

**Keywords:** rat, human, GnRH, hypothalamic regulation, effect of illumination

## Abstract

The physiology of reproduction has been of interest to researchers for centuries. The purpose of this work is to review the development of our knowledge on the neuroendocrine background of the regulation of ovulation. We first describe the development of the pituitary gland, the structure of the median eminence (ME), the connection between the hypothalamus and the pituitary gland, the ovarian and pituitary hormones involved in ovulation, and the pituitary cell composition. We recall the pioneer physiological and morphological investigations that drove development forward. The description of the supraoptic–paraventricular magnocellular and tuberoinfundibular parvocellular systems and recognizing the role of the hypophysiotropic area were major milestones in understanding the anatomical and physiological basis of reproduction. The discovery of releasing and inhibiting hormones, the significance of pulse and surge generators, the pulsatile secretion of the gonadotropin-releasing hormone (GnRH), and the subsequent pulsatility of luteinizing (LH) and follicle-stimulating hormones (FSH) in the human reproductive physiology were truly transformative. The roles of three critical neuropeptides, kisspeptin (KP), neurokinin B (NKB), and dynorphin (Dy), were also identified. This review also touches on the endocrine background of human infertility and assisted fertilization.

## 1. Introduction

It has long been known that anterior pituitary hormones regulate the sexual activity of various species. About a hundred years ago, researchers recognized that external illumination also determines sexual rhythm in various species, including fish, birds, ferrets, and mice [1,2,3,4,5,6,7,8,9,10,11,12,13]. Other researchers [14,15,16,17] have emphasized that interoceptive neural signals from the uterine and oviduct affect the sexual condition. Marshall and Bowden [18] described the estrous cycle in the rat and proposed that external environmental factors affecting the estrous cycle act through the hypothalamus. In 1930, Popa and Fielding demonstrated the vascular link between the pituitary and the hypothalamus [19]. Green and Harris [20] were the first researchers to recognize that hypothalamic peptides likely reach the anterior pituitary via the hypophyseal portal system, regulating the secretion of anterior pituitary hormones. Harris was primarily interested in hypothalamic–pituitary–gonadal control. In a monograph published in 1955 [21], he summarized all data available at that time on the neural control of the pituitary gland. In 1962, Szentágothai, Flerkó, Mess, and Halász [22] presented “a detailed anatomical and histological analysis of the hypothalamo-hypophyseal complex, with due regard to the interests of the experimenters (stereotaxic atlas, Golgi architecture, synaptology, hypothalamic pathways, blood circulation, and innervation of pituitary gland)” (quote from Akadémia Kiadó, Budapest, Hungary). The hypothalamic–anterior hypophyseal connection was named the tuberoinfundibular system. Over the next 50 years, many reviews and books were published [23,24,25,26,27,28,29,30,31,32,33,34]. One of the best summaries was written by Neena B. Schwartz (1926–2018) in 2005 [28]: “The crucial participation of hormones in reproduction and fertility is the most complicated story in endocrinology, because it involves several organ systems, gametes as well as hormones, two classes of receptors and intracellular signals, and a myriad of environmental factors, such as seasonal signals and, of course, the nearby presence of a conspecific carrier of the opposite gamete type. As complicated as this system is in mammals, being quite different among major classes, it is even more complex when one deals with the vast number of nonmammalian vertebrate species. This review is mainly limited to two groups of mammals: rodent, which is frequently chosen to elucidate the basic science, and the primates, which are clearly of primary importance for addressing clinical problems”.

In the present work, we review the development of our knowledge about the neuroendocrine regulation of ovulation in rats and describe the hormones, neuropeptides, and neurotransmitters involved in this regulation, as well as the molecular background of events, as described in recent decades.

## 2. Blood Supply of the Pituitary Gland

**Figure 1:**
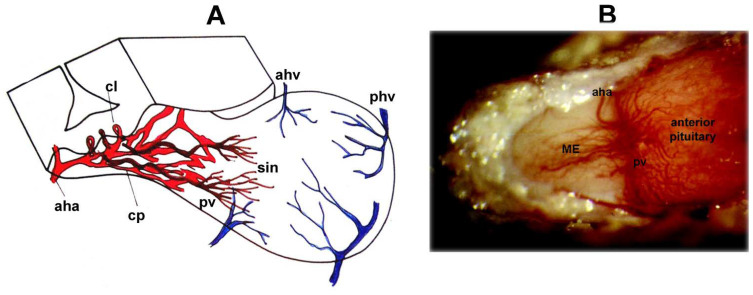
(**A**) Schematic three-dimensional view of the blood supply of the ME and anterior pituitary. Abbreviations: aha, anterior hypophyseal artery; ahv, anterior hypophyseal vein; cl, capillary loops; cp, capillary plexus; phv, posterior hypophyseal vein; pv, portal veins; sin, sinusoids. From (**B**) High-power view through a dissecting microscope of the hypophyseal portal vessels on the surface of the pituitary stalk (left) of an anesthetized rat. The portal vessels (veins) arise from the primary capillary bed on the ME (pink area to the left) and fan out over the anterior pituitary gland (right) at the pituitary stalk junction to the right. The tuberoinfundibular artery, a branch of the superior hypophyseal artery, can be seen arching across the top of the stalk–pituitary junction, where it enters the anterior pituitary gland. This artery passes through the anterior pituitary gland to supply arterial blood to the neurohypophysis. Abbreviations: aha, anterior hypophyseal artery; pv = portal vein; aha = anterior hypophyseal artery. Figure 1B is from Fink. Neural control of the anterior lobe of the pituitary gland (pars distalis). Chapter 5. In: Handbook of Neuroendocrinolgy. Eds: G. Fink, D.W. Pfaff, and J.E. Levine, pp. 97–137, 2012 [31]. ©Elsevier Inc. Amsterdam.

Over the past century, many research groups have studied the hypophyseal portal system using very different methods, such as injection with India ink, fluorescent gelatin, neoprene latex, Berlin blue, staining of red blood cells in postmortem samples, and immunostaining [35,36,37,38,39,40,41,42,43,44,45,46,47,48,49,50,51,52]. This blood supply is very special and crucial for the neurohumoral regulation of the anterior pituitary gland. The superior (anterior) hypophyseal arteries become capillaries at the border of the ME and tuberal part of the pituitary gland. This dense capillary network is called the mantel plexus. From here, capillary loops enter the layer lying above, and these capillary loops are fenestrated. Blood continues to flow from here into the portal veins, and the venous capillaries (secondary capillarization), called sinusoids, supply the anterior lobe (Figure 1). In contrast to the anterior pituitary, the posterior pituitary receives its blood supply directly from the inferior pituitary arteries.

**Figure 2:**
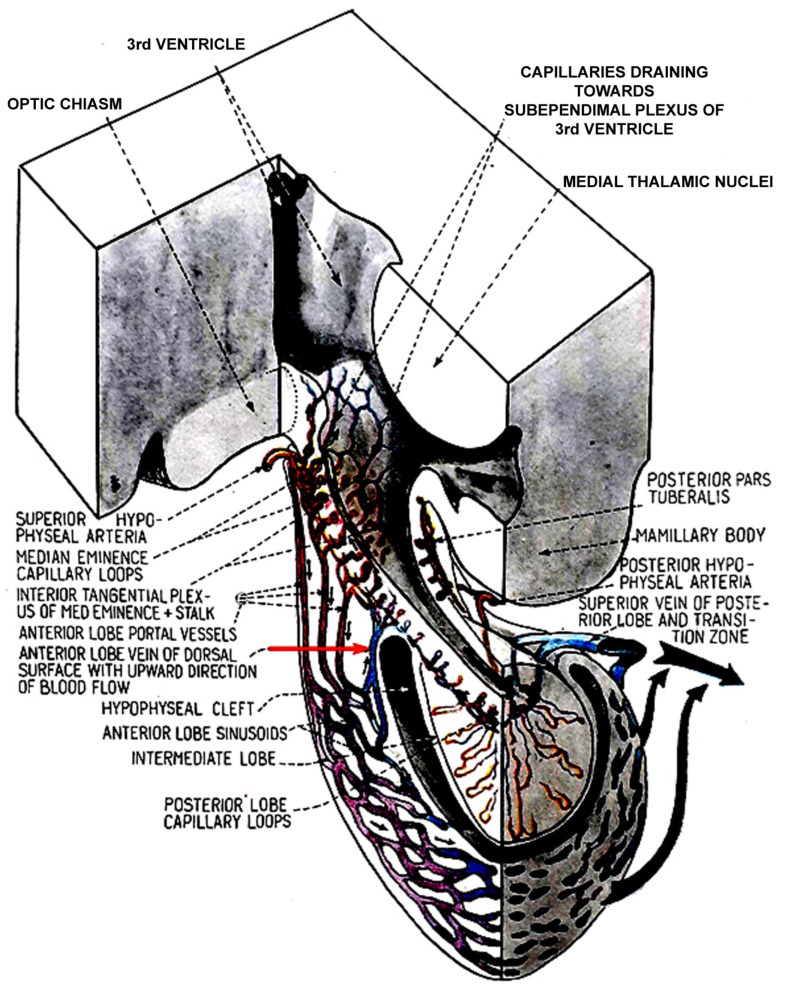
Three-dimensional drawing of the blood supply of the anterior pituitary and ME. Black arrows indicate the direction of blood flow. Red arrow shows the vein in which the blood flows upward from the anterior lobe to the capillary loops. This figure is from Szentágothai, J; Flerkó, B.; Mess, B; Halász, B. Hypothalamic control of the anterior pituitary. An experimental-morphological study, 1968 [22]. Akadémiai Kiadó, Budapest. With permission of the copyright holder: Klára Szentágothai, Budapest, Hungary.

Researchers have realized that nerve endings from the hypothalamus are not present in the anterior lobe. Therefore, it was hypothesized that the brain regulates the function of the anterior lobe through neurosecretion discharged into the portal vessels [20]. Furthermore, Popa and Fielding [19], as well as Harris [21], recognized that some blood flows back to the hypothalamus through a network of subependymal vessels. Later, other researchers confirmed this observation [22,42,48,49,50,51,52]. Török and Szentágotai [22,42] examined live dogs and cats. The two carotids of anesthetized animals were injected with different dyes, and blood flow was observed from the sub-temporal side. Viewed from this side, blood flow was definitely directed from the ME to the anterior lobe of the pituitary gland. After separating the pituitary cleft on the dorsal surface of the anterior lobe, a large number of vessels could be observed in which the blood flow was directed downward; however, in a small number of vessels, the blood flow was directed upward. After removal of the posterior lobe, blood could not flow into the posterior pituitary, but upward flow was further observed. It was concluded that the blood from here was drained into the capillaries of the infundibular recess of the third ventricle. These results suggest that the observed retrograde blood flow from the anterior pituitary to the hypothalamus does indeed exist (Figure 2).

Mezey and her co-workers [49] used a different approach to detect pituitary–hypothalamic retrograde blood flow. ^3^H-adrenocorticotropic hormone (ACTH) analog was injected into the pituitary gland of intact and pituitary stalk-sectioned rats, and the regional distribution of radioactivity in the brain was measured. They found that when ^3^H-ACTH was injected on the first day after the stalk section, the radioactivity was markedly lower in the hypothalamus than in control rats, and it was similar to controls when the ^3^H-ACTH was injected eight days after the stalk section. This observation clearly indicates that the transected vessels regenerated eight days after disruption and some blood again flowed to the hypothalamus.

In the 1980s, other methods were developed to investigate blood flow in living animals. In addition to radioactive labeling, an accepted method became the use of fluorescent polystyrene microspheres and nanoparticles. Monnet and co-workers [53] investigated the regeneration of portal vessels after complete and partial interruptions. They hypothesized that, “partial interruption of the portal vessels could provoke the establishment of a direct arterial blood supply (arteriogenesis)”. In the first experiment, polystyrene microspheres with a diameter of 15 µm were injected into the left ventricle of the heart of control rats.

Microspheres were observed in the first capillary plexus in the ME, pituitary stalk, and infundibular process. However, due to their large diameter, the microspheres failed to pass through this capillary plexus—they were trapped and did not reach the portal veins and the anterior lobe of the pituitary gland. In the second experiment, lesion of the long portal vessels did not result in arteriogenesis. However, in an extrasellar position, the anterior pituitaries—transplanted under the kidney capsule—became vascularized by direct arterial blood supply, and microspheres injected into the left ventricle of the heart appeared in the pituitary gland. In the case of in loco pituitaries, arteriogenesis was also observed when tumorigenesis was induced by 17β-estradiol (E2)-treatment. Elias and Weiner [54] treated Fischer 344 (extremely sensitive to E2) and Sprague-Dawley rats with E2, implanted subcutaneously in a Silastic capsule. A few weeks later, microspheres with a diameter of 15 µm were injected into the left ventricle of the heart. E2-induced elevation in pituitary weight and blood prolactin (PRL) levels were extremely high in Fischer 344 and moderately high in Sprague-Dawley rats. In control rats, microspheres did not reach the anterior lobe because they were trapped in the primary capillary plexus. Vascular filling revealed that new arteries entered the anterior pituitary of E2-treated rats, bypassing the portal circulation. These arteries derived from the general circulation; therefore, their blood contained lower levels of dopamine than the portal vein. The lack of inhibition of PRL due to low dopamine synthesis resulted in the formation of prolactinoma, i.e., development of a tumor (tumorigenesis) and consequential arteriogenesis in the anterior pituitary.

Researchers emphasize that the normal hypophyseal portal system is very important for normal functioning of the pituitary gland. In the middle of the last century, intensive research work was carried out to explore the development of the pituitary gland. The pituitary gland develops from two sources, the stomodeal Rathke’s pouch (entoderm) and the infundibular process of the prosencephalic vesicle (neuroectoderm). These two anlages lie together without mesoderm. In rats, the secondary capillary plexus penetrates the pituitary gland by embryonic day (Ed) 15.5 [55,56,57,58,59]. Thliveris and Currie [57] perfused human fetuses from the first trimester of pregnancy with silicone rubber. As early as 11.5 weeks of pregnancy, well-developed impregnated vessels were observed in the ME, the infundibular stalk, the pars tuberalis, and the pituitary gland. When Trandafir and co-workers [58] investigated human fetuses from the third trimester of pregnancy, the vessels resembled their adult form; however, their numbers were less. 

**Figure 3:**
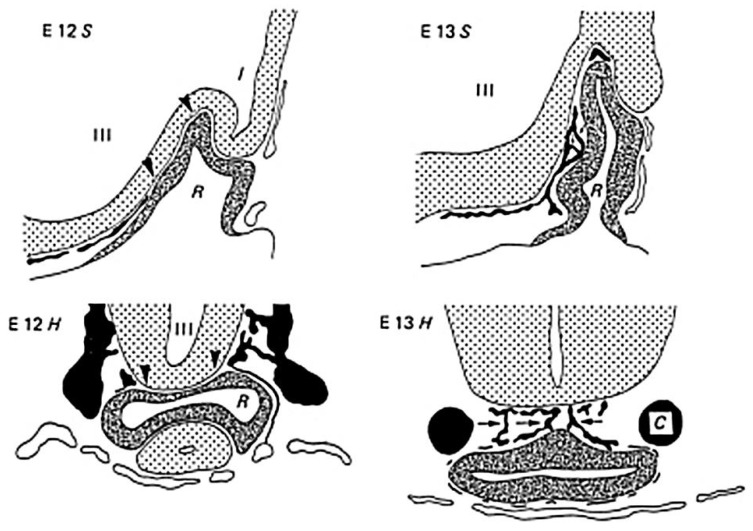
Schematic drawing illustrating the origin of the adenohypophyseal portal vessels in the sagittal (S) and horizontal (H) planes. At day 12 (Ed 12), the anterior contact zone (between the arrowheads) was free of vessels. At day 13 (Ed 13), the primary portal vessels (arrows indicate them) appeared in the separated contact zone. The empty vessels represent the anlage of the inter-cavernous sinus, which drains adenohypophyseal vessels into primary head veins. Abbreviations: R, Rathke’s pouch; I, infundibular process; III, third ventricle; C, internal carotid artery; S, sagittal section; H, horizontal section. This figure is from Szabó, K. Origin of the adenohypophyseal vessels in the rat. *J. Anat.* 1987, 154, 229–235 [60]. **©** 2024 John Wiley and Sons Inc.

Szabó [60] perfused fetuses of Sprague-Dawley rats on Ed 11, 12, and 13 with Indian ink. On the 11th day, the hypothalamic anlage received its blood supply from the primitive maxillary arteries. On Ed 12, vessels from the hypothalamic capillary network grew around Rathke’s pouch. The primary portal veins were visible at this stage. Rathke’s pouch remained in contact with the anlage of the infundibular process (posterior lobe), but it separated from the stomodeal epithelium. At this stage, the anterior surface of Rathke’s pouch (anterior lobe) was free from vessels. On Ed 13, after the separation of the anterior wall of Rathke’s pouch from the diencephalon, the primary portal veins were present and the hypothalamo–adenohypophyseal portal circulation was ready to function. The surface of the anterior lobe was covered by a capillary network originating from the vessels covering the prospective hypothalamus. These hypothalamic arteries came from the maxillary arteries, and some vessels were derived from the carotid arteries. Later, the primitive maxillary arteries diminished in size. The venous blood flowed into the inter-cavernous sinus and the primitive maxillary veins (Figure 3). The author stated that “the present observations clearly demonstrate that the adenohypophyseal vessels are derived only from the vascular network of the diencephalon, without contribution from the pharyngeal vessels, and that the portal vascular bed develops as a single entity”.

## 3. Structure of the ME

**Figure 4:**
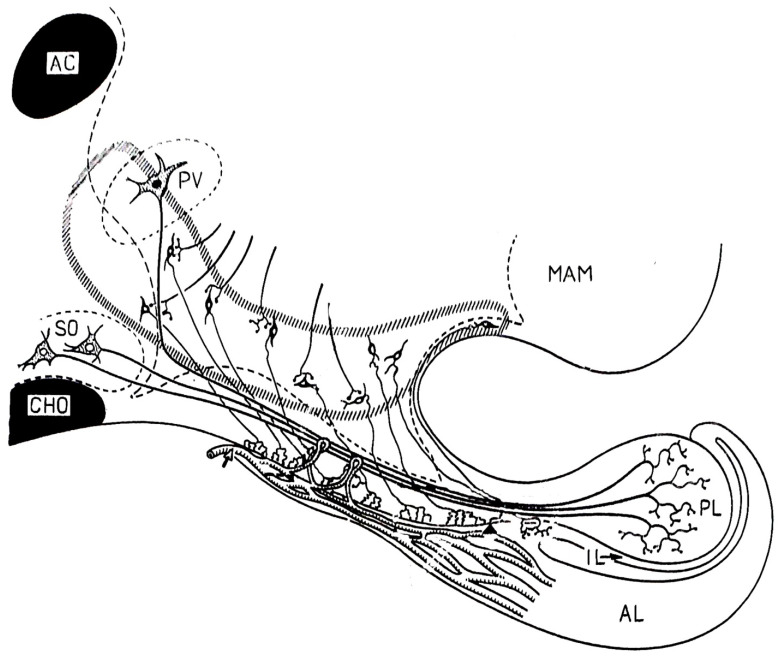
Semi-diagrammatic drawing of a midsagittal section of the hypothalamus, showing arrangement of the coarse-fiber supraoptico–neurohypophyseal tract and the fine-fiber tuberoinfundibular tract. The former originates from the large cells of the supraoptic nucleus (SO) and is joined by similar fibers arising from the paraventricular nucleus (PV). Its fibers can be traced to the posterior lobe (PL). This tract is crossed by the fine axons of the tuberoinfundibular tract arising from small nerve cells that are situated in a halfmoon-shaped area (surrounded by the hatched line) immediately beneath the walls of the third ventricle (surrounded by the dashed line). This is the so-called “hypophysiotropic area”. The fine axons of this tract terminate exclusively in the surface zone (zona palisadica) of the ME and of the most proximal part of the stalk. Zona palisadica abruptly comes to an end where the last portal veins detach themselves (straight short arrowhead) from the primary vascular plexus covering the outer surface of the ME and the stalk. The coarse fibers of the supraoptico–hypophyseal tract are seen to emerge from the dense tangle with the crossing fine fibers and to continue their way to the posterior lobe. Empty arrow indicates the superior hypophyseal artery. Abbreviations: AL, anterior lobe; AC, anterior commissure; CHO, optic chiasm; IL, intermediate lobe; MAM, mammillary body; PL, posterior lobe; PV, paraventricular nucleus; SO, supraoptic nucleus. This figure is from Szentágothai, Flerkó, Mess, Halász. Hypothalamic control of the anterior pituitary. An experimental-morphological study, 1968 [22]. Akadémiai Kiadó, Budapest. With permission of the copyright holder: Klára Szentágothai, Budapest, Hungary.

**Figure 5:**
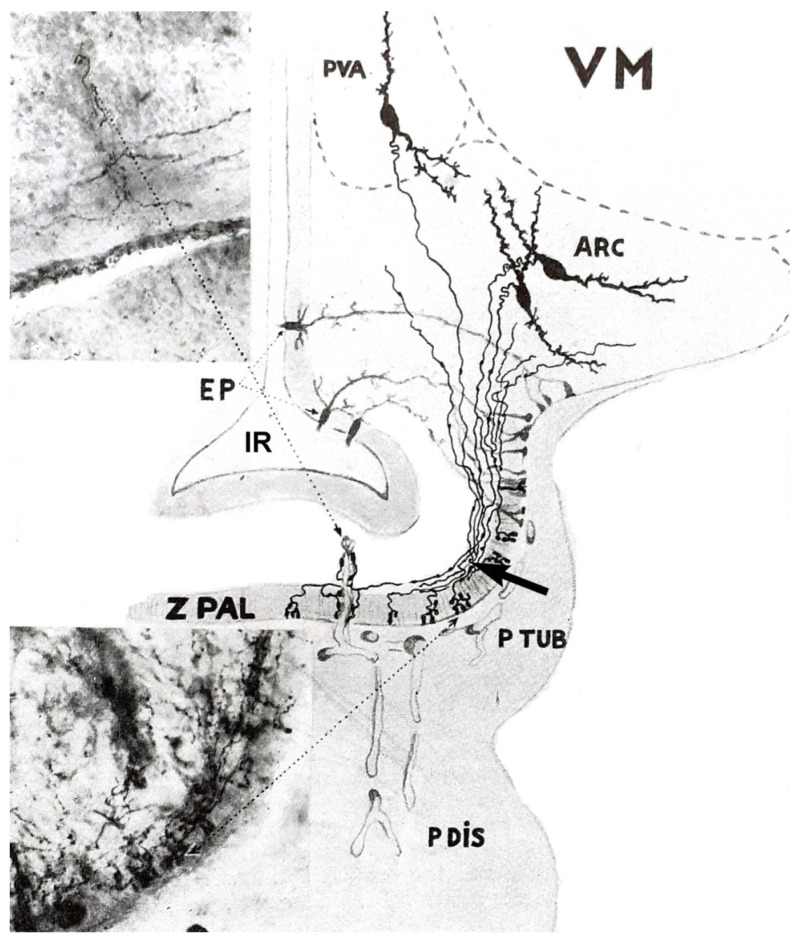
Semi-diagrammatic drawing of the tuberoinfundibular neuron system in the frontal section of the hypothalamus. Fine axons of small neurons situated in the arcuate and ventral part of the anterior periventricular nucleus enter the stalk through the lateral lip of the transition zone between the median eminence and infundibular process. The predominant direction of this tuberoinfundibular tract is transverse (black arrow). The fibers terminate partly around capillary loops of the median eminence and the proximal stalk. See the upper inset microphotograph (double-dotted arrow). Their larger part terminates in the outer zone (zona palisadica) of median eminence and the proximal stalk, i.e., on the surface, having immediate contact with the pars tuberalis. See the endings in the lower inset microphotograph (double-dotted arrow). Abbreviations: ARC, arcuate nucleus; EP, ependymal cells; IR, infundibular recess; P DIS, pars distalis; PVA, anterior periventricular area; P TUB, pars tuberalis; VM, ventromedial nucleus; Z PAL, zona palisadica. This figure is from Szentágothai, J., Flerkó, B., Mess, B., Halász, B. Hypothalamic control of the anterior pituitary. An experimental-morphological study, 1968 [22]. Akadémiai Kiadó, Budapest. With permission of the copyright holder: Klára Szentágothai, Budapest, Hungary.

**Figure 6:**
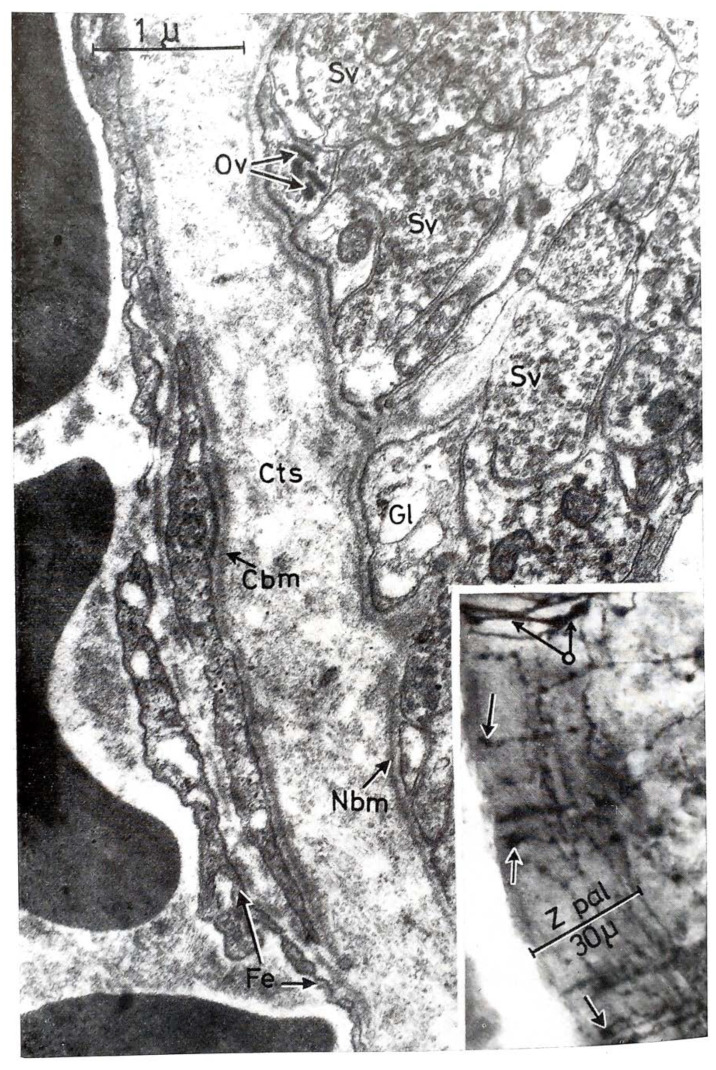
Electron micrograph of the median-eminence surface zone (zona palisadica) from an adult rat. Capillary of primary plexus containing red blood cells and lined by fenestrated endothelium can be seen on the left. A thin basement membrane of the capillary borders a narrow connective tissue space containing collagenous filaments. This tissue space is bordered at the opposite side, seen on the right, by the basement membrane of the nervous tissue. The zona palisadica is nothing but a massive layer of densely packed nerve endings, containing mostly synaptic vesicles of ordinary size and a few larger osmiophilic vesicles. The latter are rare in the most superficial zone of the nerve endings, but they became more abundant in the deeper strata. Between the nerve endings of the tuberoinfundibular tract, there are few glial end-feet, belonging mainly to ependymal fibers. The inset in the right corner shows a Golgi picture of the zona palisadica via the coarse glia end-feet (ringed arrows), the preterminal parts of tuberoinfundibular nerve fibers that run transversally in parallel to the surface, and some nerve endings (arrows). It is obvious on the scales how narrow a part of the zona palisadica is shown in the electron microphotograph. Abbreviations: Cbm, capillary basement membrane; Cts, connective tissue space; Fe, fenestra; Gl, glia end-foot; Nbm, nerve basement membrane; Ov, osmophilic vesicles; Sv, synaptic vesicles; Z, pal zona palisadica. This figure is from Szentágothai, J.; Flerkó, B.; Mess, B.; Halász, B. Hypothalamic control of the anterior pituitary. An experimental-morphological study, 1968 [22]. Akadémiai Kiadó, Budapest. With permission of the copyright holder: Klára Szentágothai, Budapest, Hungary.

Bargmann and Scharrer [61] recognized that the posterior pituitary hormones are produced in large hypothalamic magnocellular (supraoptic and paraventricular) neurons. Through the internal zone of the ME, the axons of these neurons enter the posterior lobe of the gland and release their contents into the general circulation. This pathway is called the supraoptic–paraventricular–hypophyseal magnocellular system. During this period, Green and Harris [20] assumed that the anterior pituitary is regulated by hypothalamic peptides that reach the organ via the hypophyseal portal system. A very important step was the description of the morphological basis of Harris’s theory. Many researchers observed fibers in the so-called “palisadic zone” of the ME, but it was not clear whether nerve terminals or glia end-feet form a network. In 1962, Szentágothai and his co-workers [62] described the tuberoinfundibular system. In their experiments, silver impregnation (the Golgi technique) was used. With meticulous work, they described a very dense grape-like nerve terminal layer of about 30 µm thickness. The two important systems in a sagittal section and a frontal section are illustrated in Figure 4 and Figure 5, respectively. Two years later, Szentágothai and Halász’s electron microscopic study [63] clarified the difference between the fibers of the supraoptic–paraventricular–magnocellular and tuberoinfundibular parvocellular pathways (Figure 6).

According to light and electron microscopic investigations, the ME has a layered structure [62,63,64,65,66,67,68]. This structure contains nerve terminals, glial cells, and capillaries, but there are no synapses. The ME is the area where nerve endings release hypophysiotropic substances into the capillaries of the primary plexus. The ME is a neuroendocrine transducer that conveys information in the form of neuropeptides and neurotransmitters from the hypothalamus into the pituitary gland. The ME is also the interface between the cerebrospinal fluid of the infundibular recess and the blood of the primary plexus, where specialized ependymal cells transport hypophysiotropic substances from infundibular recess into the portal blood.

**Figure 7:**
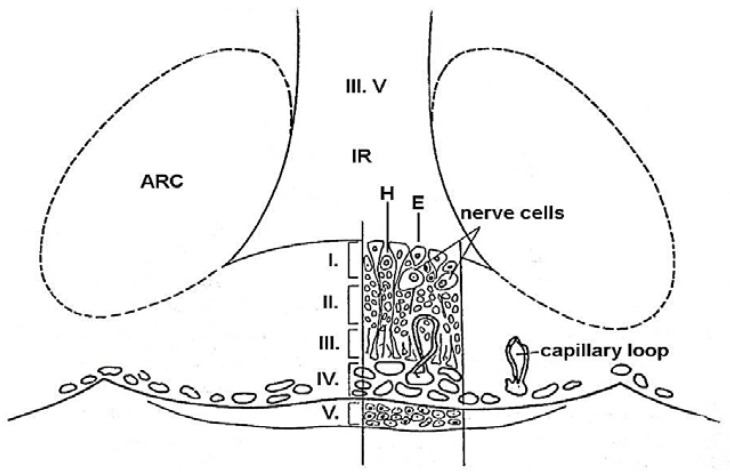
Schematic illustration of ME in rats. Abbreviations: III V, 3rd ventricle; ARC, arcuate nucleus; E, ependyma; H, hypendyma; IR, infundibular recess; I, ependymal and hypendymal cells; II, internal zone; III, external zone (palisadic); IV, mental plexus; V, tuberal part of the anterior pituitary.

The ME is composed of five layers (Figure 7):

Layer I. Its most inner surface facing the cerebrospinal fluid of the third ventricle is composed of ependymal and hypendymal cells. Nerve cells were also identified below the ependymal layer [69,70].

Layer II. The next layer contains coarse axons of the magnocellular neurosecretory neurons. It is generally called the internal zone. Only coarse fibers run through this layer.

Layer III. The external or palisadic zone contains nerve endings of fine fibers of mainly hypothalamic origin and glial end-feet. It is the termination of the tuberoinfundibular system.

Layer IV. This is the previously mentioned mental plexus. From here, capillary loops penetrate the external zone where the neurovascular contact surface is found. This surface is 26% larger in female rats than in males [69]. Another functionally important compartment of the ME is formed by a network of fenestrated capillary loops [70]. These long loops are mainly surrounded by ME tanycyte terminals [71] and neurosecretory endings of neuroendocrine neurons (Figure 5).

Layer V. The outer surface of ME is covered by the tuberal part of the pituitary gland. It contains secretory and stellate cells.

The ME is one of the circumventricular organs where the blood–brain barrier (BBB) is missing [72,73]. The BBB is a dynamic, semipermeable system in the cerebral micro-vessels. It separates the bloodstream from the extracellular fluid of the brain. The barrier is composed of endothelial cells, basement membrane, pericytes, and astrocyte end-feet. The endothelial cells are connected by tight junctions. In contrast, in the circumventricular organs, such as the ME, the usual tight-junction endothelial appositions are missing, and the capillaries are fenestrated (Figure 6). The nerve endings of the tuberoinfundibular tract establish intimate contact with the fenestrated capillaries and, here, certain molecules from the blood can be taken up by the nerves, i.e., two-way blood–brain communication takes place.

## 4. Ovarian Hormones Involved in Ovulation: Estrogen (E) and Progesterone (P)

In the early 1900s, significant progress was made in understanding the reproductive system, not only at the level of the central nervous system (CNS), but also at the level of the ovary. E was first described in 1923 as the hormone of the ovarian follicle [74], and six years later, P was discovered in the corpus luteum [75]. The basic function of these steroid hormones is to regulate the development and function of the uterus and mammary gland and in case of fertilization, to maintain pregnancy. 

E2 is the predominant type of E during reproductive years. In females, androgen is secreted by the theca cells in the follicle and converted into E by granulosa cells. P is secreted by both granulosa and theca cells in the follicle, and in both luteal cells in the corpus luteum [76]. Steroids exert their effect through specific receptors. It was a milestone when Jensen and his co-workers [77] discovered the E receptor in 1967. They demonstrated that E-bound receptors migrate to the nucleus, where they could stimulate gene transcription [78]. During the next 50 years, 3 different types of E receptors were identified: ERα [79], ERβ [80], and the G-protein-coupled E receptor, GPR30, which predominantly binds to intracellular membranes (endoplasmic reticulum and Golgi apparatus) [81]. Nuclear binding of this receptor was also observed [82].

Knockout (KO) animal models were used to demonstrate the significance of these receptors in reproduction. Dupont and co-workers [83] investigated E2 receptor knockout (ERKO) female mice. It was found that ERαKO animals are sterile and ERβKO animals are sub-fertile. However, E2 effects were unaltered when GPR30 was knocked out, and in endothelial cells (ECs) derived from ERα/ERβ combined-KO mice, E2 failed to bind and activate cAMP, extracellular signal-regulated kinase (ERK), or phosphatidylinositol 3-kinase, nor did it stimulate DNA synthesis [84]. In lower vertebrates, such as zebrafish, GPR30 plays an important role in reproduction. GPR30 affected ovarian follicle development via regulation of vitellogenesis and endothelial growth factor expression, which had an important role in the folliculogenesis. In zebrafish, the lack of GPR30 reduced fertility by about 40% [85]. Since the E-positive feedback is one of the very important events to initiate ovulation, the ERs play a crucial role in reproduction [86].

P is involved in ovulation, implantation, and pregnancy. It is produced by the lutein cells of the corpus luteum during the luteal phase of the reproductive cycle and by the syncytiotrophoblast of the placenta during pregnancy. P circulating in the bloodstream is bound to cortisol-binding globulin and serum albumin. P receptors were discovered about 50 years ago. Three isoforms were identified, P receptor A (PRA), PRB, and PRC: all are nuclear receptors [87]. Normally, all three receptor isoforms are involved in the maintenance of pregnancy in mammals [88]. Their significance was explored with the help of KO animals. PRA-KO induced severe abnormalities in the functioning of the ovaries and the uterus, leading to female infertility. PRB knockout (PRBKO) mice have shown that ablation of PRB does not affect ovarian, uterine, or thymic responses to P, but rather reduces mammary ductal morphogenesis [89,90]. 

Thirty years ago, Baulieu and Robel [91] demonstrated that steroids are also produced by glial cells in the brain. Steroid hormones circulating in the blood are precursors for the synthesis of neuro-steroids. These molecules regulate behavioral activities, stimulate neurons, and modulate the function of gamma aminobutyric acid (GABA) A receptors through rapid, non-genomic actions [92].

In the mid-1900s, several methods were developed to determine the presence of E and P in the organism. Papanicolau and Traut [93] recognized that the stage of the estrous cycle in the experimental animals could be determined by histological examination of the cells of vaginal smears. Biological tests have also been used to determine the presence of E in plasma and urine [74]. The next step was the introduction of immunoassays and liquid chromatography. Yalow and Berson [94] developed the first radioimmunoassay (RIA) for insulin, and Abraham [95] developed the solid-phase RIA for E. Rosner and co-workers [96] reviewed the methods used worldwide in the experimental and clinical practices for the measurement for E. They found that chemiluminescent immunoassays and liquid chromatography/tandem mass spectrometry-based methods are most favored nowadays. Li and co-workers [97] further developed solid-phase extraction with liquid chromatography–tandem mass spectrometry for the determination of six Es and E mimic molecules, namely, estriol, bisphenol A (BPA), E2, estrone, ethynyl estradiol, and dienestrol. Recently, Casals and co-workers [98] recommended liquid chromatography/tandem mass spectrometry (LC-MS/MS) as the most reliable method in clinical practice. 

Vitellogenin, the precursor of the yolk protein in egg-bearing vertebrates, is essential for oogenesis, embryonic development, and larval survival. One of the newest methods to reveal the E in an examined sample was recently introduced by examining the effect of E on vitellogenin. mCherry fluorescent protein-encoding region was built downstream to the promoter of the zebrafish vitellogenin-1 gene. Fragments were cloned into the construct with the Gateway site-specific recombination technique. Normally, vitellogenin genes are only expressed in adult females, and not at all or at very low levels in larvae and males. However, these genes are expressed in the presence of E. This method also allows for quantitative determination of E in the given sample [99].

Methods similar to E determination have also been developed for determination of P, such as RIA [100], enzyme-linked immunosorbent assays (ELISA) [101], and isotope dilution liquid chromatography/tandem mass spectrometry [102]. 

## 5. Anterior Pituitary Cell Composition, Pituitary Hormones Involved in Ovulation: LH, FSH, and PRL

Joseph Lieutaud was the first anatomist (1703–1780) to recognize the portal system of the pituitary gland and to describe it in an anatomy book [103]. As mentioned above, the anterior pituitary (adenohypophysis) derives from the stomodeum. It is composed of three parts: pars distalis, pars tuberalis, and pars intermedia. In rats, on Ed 12, vessels from the capillary network of the hypothalamic surround Rathke’s pouch [59,60]. 

Over the past three decades, the number of articles and books dealing with the molecular basis of pituitary vascularization and the development of the pituitary cell composition has tremendously increased [104,105,106,107,108,109,110,111]. Signaling molecules and transcriptional factors are involved in the differentiation of anterior pituitary cell lineages. In situ hybridization techniques and gene-modified (knockout, knockdown, and transgenic) animals were usually used. Zhao and co-workers [108] described the role of the LIM-homeobox gene (Lhx3) in the differentiation of Rathke’s.

Treier and co-workers [104] discussed three successive phases of signaling events involved in the development of the pituitary gland in mice. The ventral diencephalon, which expresses signal molecules (bone morphogenetic protein (BMP4), fibroblast growth factor protein (FGF) 8/10/18, and wingless protein (Wnt5)), directly contacts the oral ectoderm (which includes the hypophyseal placod) and gives rise to Rathke’s pouch. In the absence of BMP4, Rathke’s pouch formation occurs, but pituitary cell lineages fail to develop, except for a few corticotropes (C). Sonic hedgehog protein (Shh) is expressed throughout the oral ectoderm, except in Rathke’s pouch, which creates a boundary between two ectodermal domains of Shh-expressing and Shh-non-expressing cells. Opposing dorsal BMP4/FGF and ventral BMP2/Shh gradients mediate proliferative and positional signals by regulating combinatorial patterns of transcription factor gene expression. Pituitary-specific transcription factor (Pit1) is induced on Ed 13.5 in the caudomedial region of the pituitary gland of mice, which gives rise to somatotropes, lactotropes, and thyrotropes. Rostral tip thyrotropes are Pit1-independent. C and gonadotropes (G) are differentiated in the most ventral part of the gland. The dorsal region of Rathke’s pouch becomes the intermediate lobe containing melanocytes. The infundibulum grows downward and eventually becomes the posterior lobe. The further development of Gs is regulated by steroidogenic factor-1 (SF-1). SF-1 is a nuclear receptor that also plays a key role in gonad development. Disruption of SF-1 leads to gonadal agenesis, defective differentiation of the ventromedial hypothalamus, and impaired expression of luteinizing hormone (LH)β, follicle-stimulating hormone (FSH)β, and GnRH receptor in the pituitary gland. *SF-1*^/−^ (knockout) pituitary phenotype results in sterile mice with hypoplastic gonads. GnRH deletion in the hypothalamus results in decreased LHβ, FSHβ, ACTH, and PRL cells in the pituitary, and GnRH-R mutation in the anterior pituitary induces a dramatic reduction in the numbers of FSH- and LH-producing Gs.

Zhao and co-workers [108] used pituitary-specific SF-1 KO mice to demonstrate the role of this factor. They focused on the development of Gs. KO mice were generally viable, but they were sterile and did not show normal secondary sex characteristics. Zhu and co-workers [109] also reviewed the signal molecules and transcriptional factors that regulate the development of Rathke’s pouch and pituitary cell types. These researchers described step-by-step when and which transcription factor or signal molecule intervenes in controlling the further development of the pituitary gland. They mainly focused their description on somatotropes and lactotropes.

The abovementioned results clearly show that the pituitary is not a randomly organized collection of cells. However, in recent years, it has been established that, “pituitary cells are highly organized networks of cells that communicate with each other allowing the gland to quickly adapt to changing physiological demands” [110]. Himes and Raetzman [111] demonstrated the importance of two early-acting Noth signal molecules, Hes1 and Prop1. In the absence of these molecules, the cells are trapped in the Rathke’s pouch and lose their migration ability.

Vascular endothelial growth factor A (VEGFA) expression, one of the best-studied transcriptional factors, is detectable on E embryonic day 12 in rats. It is important for the normal development of the anterior lobe. Later, its expression persists in the folliculostellate cells and some hormone-producing cells. However, ectopic expression of VEGFA in the pars intermedia causes reduced expression of the differentiation markers of melanocyte-stimulating hormone (MSH) and prohormone convertase 2, and this condition increases the growth of the intermediate lobe [112,113]. VEGFA stimulates vascularization. Treatment with an anti-VEGFA antibody reduces growth of pituitary prolactinoma and serum PRL levels in a mouse model [114]. Development of the hypophyseal arteries and portal system may be abnormal in some children with hypopituitarism, but it is not clear whether this is the cause or the consequence of VEGFA expression being insufficient for normal angiogenesis. Prop1 encodes a paired-like transcription factor restricted to the developing anterior pituitary. Prop1 is required for the determination of various cell types in the anterior lobe. Prop1 mutant pituitaries express VEGFA, but they have poor vascularization, failed differentiation, and increased apoptosis [113].

Japon and co-workers [115] studied the gene expression of anterior pituitary hormones in female mice by in situ hybridization from Ed 9.5 to postnatal day 1. The common alpha-glycoprotein subunit (alpha-GSU) mRNA first appeared in the anterior wall of Rathke’s pouch on Ed 11.5 and extended to the pars tuberalis and ventromedial zone of the pars distalis on Ed 12.5. Thyroid-stimulating hormone-beta (TSHβ) subunit mRNA was expressed in the same place on Ed 14.5. The expression of both growth hormone (GH) and PRL started coincidentally on Ed 15.5. In contrast, LHβ and FSHβ subunit mRNAs were detected initially only in the ventromedial part of the pars distalis on Ed 16.5 and Ed 17.5, respectively.

Long before the molecular era, it was recognized that the anterior pituitary was composed of hormone-producing and folliculostellate cells. With the use of basic histological stains (H&E, PAS, and Orange-G), three anterior pituitary cell types could be identified: acidophils, basophils, and chromophobes. Later, the researcher realized that somatotropes and lactotropes are acidophilic, Gs and thyrotropes are basophilic, and Cs and folliculostellate cells are chromophobes. The pioneer biochemist, Choh Hao Li, in 1954 discovered that the ovine ACTH is composed of 39 amino acids [116], and human pituitary somatotropin (GH) consists of a chain of 256 amino acids [117]. Later, the other pituitary hormones were identified. PRL is composed of 199 amino acids [118]. It has several isoforms: the little, big, and big-big forms. It was shown that the little form has well-defined biological activity [119].

Thyroid stimulating hormone (TSH) [120] and the two gonadotropins, FSH and LH, are glycoproteins composed of α- and β-subunits. Alpha-subunit is common in the three hormones and made up of 92 amino acids. Beta-subunits are different. In the TSH, it is composed of 118 amino acids, in the FSH, the β-subunit is composed of 111 amino acids, and in the LH, the β-subunit is composed of 120 amino acids [121].

## 6. Hypophysiotropic Area

Parallel to progress in pituitary research, the “hypophysiotropic area” was recognized [122,123,124]. This area is located in the medial basal hypothalamus (MBH). Researchers hypothesized that the MBH produces certain factors that maintain the normal structure and function of the pituitary gland. Pituitary gland implantation experiments support this view. When a pituitary graft was placed within the hypophysiotropic area of hypophysectomized rats, animals showed resumption of normal vaginal cycles and corpora lutea formation within a few months.

Nikitovitch-Winer and Everett [125,126,127] implanted female rat pituitary glands under the renal capsule. There was damage in the cytology of the gland. After 2–3 weeks, some cell types, except gonadotropes, were detected. The pituitary gland was then removed and re-transplanted under the ME of the same animal. A few months later, striking recovery occurred and the gonadotropic function was restored in the hypophysectomized animals. The grafts in the hypothalamus were richly vascularized and cyto-differentiated into various types of anterior pituitary cells; however, only well-developed lactotropes, but poorly developed other cell types, were visible in the grafts under the renal capsule. In the 1970s, it was clearly demonstrated that hypothalamic factors can also influence the differentiation of the clonal strain of anterior pituitary cells derived from Rathke’s pouch of rats. Clonal cells were implanted under the renal capsule or in the hypophysiotropic area of hypophysectomized female rats. The latter cells differentiated into normal pituitary cell types [128]. Halász and co-workers [122,123,124] clearly demonstrated that the hypophysiotropic area, but not other parts of the hypothalamus, can maintain the tropic hormone secretion of the pituitary graft.

About a hundred years ago, in the 1930s, the releasing (liberins) and inhibiting factors (statins) were not known by the researchers. However, it was clear that various drugs acting on the CNS or electrical stimulation of the CNS could induce ovulation [129,130]. First of all, the role of the hypothalamus emerged [131]. Marshall and his co-workers [132] used a rabbit model to study the role of the hypothalamus in the induction of ovulation. Ovulation in rabbits usually occurred only after some kind of sexual excitement (heat; ovulation induced or provoked by an external stimulus during mating or just prior to mating). Into the ear vein, they injected several drugs into rabbits on heat. The drugs were known as stimulators of the CNS. Pilocarpine, escorine, acetylcholine, adrenalin, strychnine, apomorphine, β-tetrahydronaphthylamine, ergometrine, carbamylcholine hydrochloride, coriamyrinehydrochloride, and picrotoxin were administered. Only picrotoxin (0.9–1.1 mg/kg) was able to elaborate ovarian responses and ovulation. This research group also applied electrical stimulation [129,133]. They were also able to induce ovarian responses in rabbits after stimulation of the pituitary or tuber cinereum. Some follicles became very protuberant and showed some internal hemorrhage, or others became corpus luteum.

Later, rats were usually used to study the regulation of ovulation. The estrous cycle in rats was first described by Long and Evans a hundred years ago [134]. The normal cycle is composed of four days: proestrus, estrus, metestrus, and diestrus. The vaginal smears have very characteristic appearances in the different stages. Proestrus is characterized by the presence of round, nucleated, uniform cells. During estrus, there are only enucleated cornified epithelial cells, and the cell shape is irregular. During metestrus, in addition to enucleated cornified cells, many leukocytes appear. Diestrus is characterized by the polymorphonuclear leukocytes, occasionally a few epithelial cells, and prominent mucous discharge. The histological features of stages in vaginal smears, uterus, and the ovaries have recently been redescribed in detail by Ajayi and Akhigbe [135].

**Figure 8:**
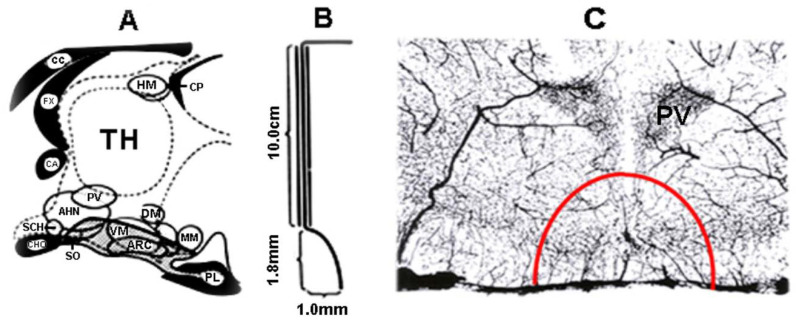
(**A**) Schematic drawing of the experimental preparation in animals with neural isolation of the hypophysiotropic area. The area cut around (hatched region) is in contact with the pituitary but is disconnected from the rest of the brain, as indicated by the heavy line. (**B**) A schematic drawing of the knife assembly used for hypothalamic deafferentation. (**C**) Blood supply of the hypothalamus in an Indian-ink-perfused animal. In the frontal section, the red line represents the knife cut. Abbreviations: AHN, anterior hypothalamic nucleus; ARC, arcuate nucleus; CA, anterior commissure; CC, corpus callosum; CHO, optic chiasm; DM, dorsomedial nucleus; FX, fornix; HM, medial habenula; MM, mammillary body; PC, posterior commissure; PL, posterior lobe; PV, paraventricular nucleus; SCH, suprachiasmatic nucleus; SO, supraoptic nucleus; TH, thalamus; VM, ventromedial nucleus. This figure is from Szentágothai,J.; Flerkó, B.; Mess, B.; Halász, B. Hypothalamic control of the anterior pituitary. An experimental-morphological study, 1968 [22]. Akadémiai Kiadó, Budapest. With permission of the copyright holder: Klára Szentágothai, Budapest, Hungary.

With the above-described experimental results, it was accepted that the MBH has a mandatory role in the regulation of the gonadotropic function. It was an amazing idea to separate the MBH from the rest of the brain. Béla Halász, a young researcher at the time when he constructed a bayonet-shaped knife (Figure 8), elaborated the deafferentation technique [124].

**Figure 9:**
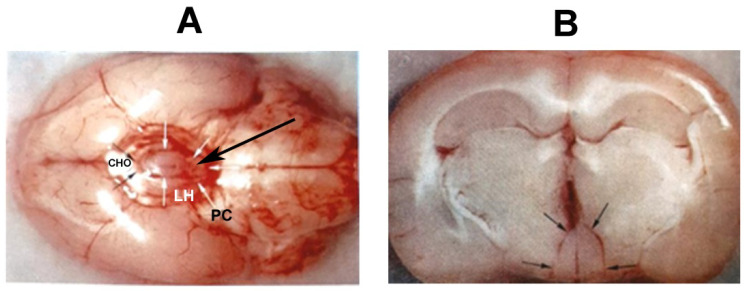
(**A**) Complete deafferentation of the hypophysiotropic area of a female rat, as seen from the base of the brain. (**B**) Extent of the deafferented region on a frontal section of the brain. White arrows indicate the cut. Large black arrow shows the pituitary gland. Abbreviations: CHO, optic chiasm; LH, lateral hypothalamus. This figure is from Szentágothai, J.; Flerkó, B.; Mess, B.; Halász, B. Hypothalamic control of the anterior pituitary. An experimental-morphological study, 1968 [22]. Akadémiai Kiadó, Budapest. With permission of the copyright holder: Klára Szentágothai, Budapest, Hungary.

The knife was fixed to the holder of the stereotaxic instrument. Through a hole in the skull roof, the knife was lowered to the base of the skull in front of the MBH. The tip of the knife faced forward. Then, it was turned 90° to the right, moved backwards, then turned 180° to the left, and then moved forward and turned to the midline. In the case of the hypophysiotropic area deafferentation, the isolated area kept its blood supply because the vessels from the circle of Willis run up parallel with the direction of the cut (Figure 8C). In Figure 9, the knife cut is seen from below on the base of the brain (A) and in a frontal section through the anterior part of the ME (B).

It has become evident that in the rats, bearing isolation of the hypophysiotropic area from the rest of the brain, the pituitary keeps its normal histology and weight. It was hypothesized that those peptides that are involved in this effect are produced inside the deafferented area. This area did not contain the suprachiasmatic (SCN) and paraventricular (PVN) nuclei and the preoptic area (PON). SCN, the “biological clock”, receives its blood supply from forward and it cannot be included in the whole isolation. Unfortunately, the rats did not ovulate; however, another spontaneous ovulator rodent, for example, guinea pig, was able to ovulate. The deafferentiated MBH in the latter species is capable of sustaining both tonic and phasic secretion of gonadotropins, necessary to maintain the normal estrous cycle and ovulation [136]. In the case of so-called “preoptic area deafferentation” (disruption of the connections of this area from forward, lateral side, and above), the adult rats kept their ability to ovulate [137]. Later, it became evident that luteinizing hormone-releasing hormone (LHRH) neurons involved in ovulation are located in the preoptic area. Partial deafferentations (anterior, posterior frontal cuts, etc.) were also carried out to study the role of different afferent pathways in the function of the MBH [138,139,140].

## 7. Discovery of Releasing and Inhibiting Hormones

The next milestone was the isolation and characterization of the hypothalamic releasing and inhibiting hormones. Their discovery was truly a transformative development in the field of neuroendocrinology. Thyrotropin-releasing hormone (TRH) was isolated and characterized in Guillemin’s laboratory from ovine [141,142], and at the same time in Schally’s laboratory from porcine [143,144] hypothalami. The characterization revealed that TRH is a tripeptide (5-Oxo-L-prolyl-L-histidyl-L-prolinamide). Two years later, LHRH was first isolated and characterized from porcine [145,146] and subsequently from ovine [147] and chicken hypothalami [148]. This peptide is composed of ten amino acids (free acid decapeptide). Schally’s research group demonstrated that not only the hypothalamic extract but also the synthetic LHRH stimulated the release of LH and FSH in young healthy male and female volunteers [149]. This observation means that the same releasing hormone has dual capability. Later, LHRH was named GnRH. The RIA was previously elaborated by Berson and Yalow in 1959 based on the idea of an immunologist, J. R. Marrack (physicochemical interpretation of antigen–antibody interactions) [150]. This method allowed the possibility to measure small amounts of hormones in blood samples or tissues.

**Figure 10:**
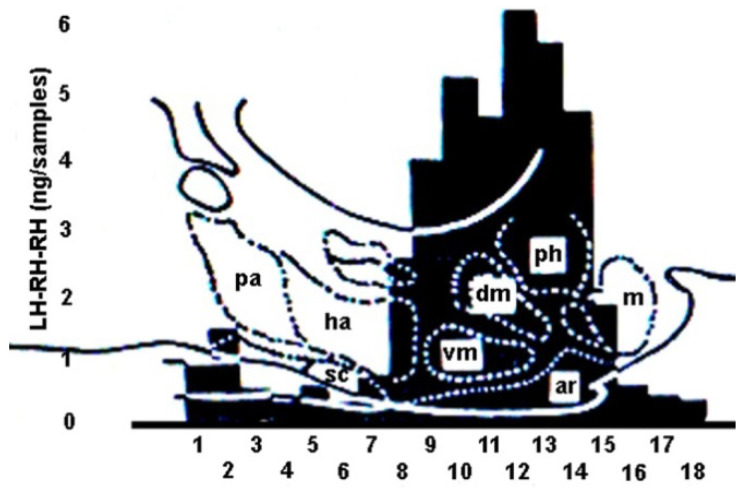
Results of assays of pooled coronal sections from three diestrus female rats. The values of LHRH are provided in nanograms (ng) per sample. Values for each sample are superimposed over a sagittal outline of hypothalamic nuclei. Sample I was taken at the level of the anterior commissure. Abbreviations: ar, arcuate nucleus; dm, dorsomedial nucleus; ha, anterior hypothalamic area; m, mammillary nuclei; pa, preoptic area; ph, posterior hypothalamic area; sc, suprachiasmatic nucleus; vm, ventromedial nucleus. This figure is from King, J.C.; Williams, T.H.; Arimura, A. Localization of luteinizing hormone-releasing hormone in rat hypothalamus using radioimmunoassay. *J. Anat*. 1975, 120, 275–288 [152]. ©2024 John Wiley and Sons Inc.

The first GnRH antibody was raised in rabbits in Schally’s laboratory by Arimura and co-workers. They elaborated the GnRH RIA in possession of the antibody [151]. Later, King and co-workers [152] investigated the GnRH levels in male and female rat hypothalamus. Here, 150 µm-thick coronal sections were taken at 18 levels. The first was rostral to the anterior commissure, the last was taken from behind the mammillary nuclei. Similar regions from five rat brains were processed for further assays. The values were analyzed individually or superimposed in a chart, as seen in Figure 10. In female rats, on the second day of diestrus, the highest GnRH level was found in the ME and the middle third of the arcuate nucleus, and the level above the pituitary stalk dramatically decreased. A moderate elevation was observed in the sections containing the preoptic area. In the GnRH levels, they also found variations depending on the sex of the animals and the stage of the female estrous cycle.

Scharrer [153] named the ME and the middle third of the arcuate nucleus the ‘final common path’, which is so important to regulate the pituitary-dependent endocrine organs and ovulation. After cutting all known inputs to this region with the Halász knife [122], tonic (but not phasic) release of pituitary gonadotropins is maintained. Previously, it was demonstrated that ovulation is evoked by stimulation of the arcuate–ME region [154]. The same stimulation caused elevation of plasma LH, and it seems reasonable to suppose that this LH elevation is a consequence of GnRH release from the arcuate–ME region. Browstein and his co-workers [155] clearly showed that 70–90% of GnRH derives from outside of the hypophysiotropic area. When a specific antibody was available, using immunohistochemistry, other researchers provided better insight into the organization of the GnRH system. King and co-workers [156] described the GnRH system using an unlabeled antibody peroxidase–anti-peroxidase (PAP) immunohistochemical method. Barry and co-workers [157] used a fluorescent-labeled antibody, and Baker and co-workers [158] and Sétáló and co-workers [159] also used PAP methods. Merchenthaler and co-workers [160] combined immunohistochemistry with deafferentation techniques. The analysis of the data revealed that, “(i) GnRH axons of the MBH of the rat originate from extrahypothalamic areas and approach the ME through the preoptic-infundibular GnRH tract; (ii) axons from the lateral fascicles of this tract turn toward the ME under oblique angle, consequently, a paramedian sagittal cut causes primary degeneration of the GnRH terminals only behind the rostral level of the cut, and diminution of these terminals will last even behind the caudal end of the cut; (iii) the tuberoinfundibular tract of the rat does not contain GnRH axons (but it contains other releasing and inhibiting hormones!), and (iv) a certain percentage of the GnRH axons in the ME originates from the contralateral side of the brain”. Several researchers have studied the GnRH system using immunohistochemical methods in control and various rat models (ovariectomized and E2-treated, and neonatally androgen sterilized) [161,162]. They observed that the GnRH neuronal system is a very dynamic system. It shows changes depending on the condition of the endocrine system, such as gonadectomy, different stages of the estrous cycle, and during the preovulatory LH surge on the day of proestrus afternoon. GnRH is not present in peripheral blood but is present in the portal vein. In rats [163] and rabbits [164], portal blood was collected, and GnRH was measurable. The relation between GnRH and LH required simultaneous measurements of GnRH in portal blood and LH in the peripheral blood. Using sequential samples of stalk blood from rhesus monkeys, Carmel and co-workers [165] demonstrated that the GnRH concentration fluctuates in pituitary portal blood, i.e., GnRH showed pulsatile release in monkeys [166].

**Figure 11:**
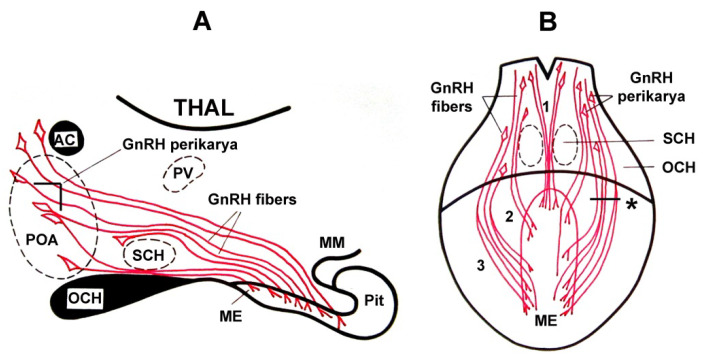
Schematic illustration of the GnRH neuronal system in a sagittal section of the hypothalamus (**A**) and as seen from the base of the brain (**B**). Cell bodies are located in the medial preoptic and in the medial septal areas. 1: Median bundle. 2: Medial bundle. 3: Lateral bundle. Abbreviations: AC, anterior commissure; OCH, optic chiasm; Pit, pituitary gland; ME, median eminence; MM, mamillary body; PO, preoptic area; PV, paraventricular nucleus; SCH, suprachiasmatic nucleus; THAL, thalamus; *, retrochiasmatic gate.

It became evident that a pretreatment of adult animals with colchicine (a drug, which inhibits the rapid axon transport and keeps the hormone in its production site, i.e., in the cell bodies [167]) improves the detection of GnRH cell bodies. When the fixation of tissues, the titer, and specificity of antibodies were carefully controlled and intensification techniques and indirect immunostaining were introduced, the exploration of GnRH neurons improved tremendously [168,169]. Immunohistochemistry combined with colchicine pretreatment and tract-tracing techniques revealed that about 70% of the GnRH neurons, located in the septo-preoptic-anterior hypothalamic area, projected to the ME. The rest of the neurons projected to other regions [170]. The distribution of GnRH fibers in the ME was also studied by Anthony and co-workers [171]. In rats, the GnRH fibers were located in the external zone, mainly in its lateral parts. In other species, the immuno-positive fibers were seen in both external and internal zones as well. In prepubertal rats, the cell bodies are easily stainable without colchicine pretreatment. The staining is most intense before the onset of puberty, which occurs in the Sprague-Dawley rats 30–36 days after birth [172]. Figure 11 schematically shows the GnRH pathway. The pathway has three bundles at both sides: median, medial, and lateral bundles. The lateral bundle enters the ME through the “retrochiasmatic gate”, as described by Palkovits [173].

The GnRH system is composed of a relatively small number of the cells. Wray and Hoffman [174] carried out a quantitative examination in postnatal rats. They found that about 1300 immunoreactive GnRH cells existed within the forebrain at all ages, both in male and female rats. These observations were confirmed and extended by Merchenthaler and co-workers [175]. Postnatal prepubertal rats were investigated using the silver–gold intensification method. GnRH-immunoreactive perikarya were found in the anterior hypothalamus and in extrahypothalamic regions as well: in the olfactory bulb and tubercle, vertical and horizontal limbs of the diagonal band of Broca, medial septum, medial preoptic, and suprachiasmatic areas, anterior and lateral hypothalamus, and different regions of the hippocampus (indusium griseum and Ammon’s horn). In addition to the known GnRH pathways (preoptico-terminal and preoptico-infundibular), GnRH immunoreactive fibers were found in the periventricular stria terminalis, stria medullaris thalami, fasciculus retroflexus, medial forebrain bundle, stria longitudinalis medialis and lateralis, hippocampus, periaqueductal gray matter of the mesencephalon, and extracerebral regions, such as the lamina cribrosa, nervus terminalis, and its associated ganglia.

After the discovery of GnRH, its receptors were soon identified [176]. Knowledge of the structure, distribution, and expression of GnRH receptors (GnRHR) is important to explore its role in the gonadotropic functions. GnRHR is a 60 kDa G-protein-coupled receptor, with seven transmembrane domains. It has two forms: GnRhRI and GnRHRII. GnRHRs are widely distributed in mammals. The GnRHR assay (equilibration with iodine-labeled ligand ([^125^I]iodo-[D-Ala^6^]des-Gly^10^-GnRH N-ethylamide) [177] revealed that GnRHRI has a neuroendocrine role and it is expressed on the surface of pituitary gonadotropic cells. GnRHRII influences sexual behavior. GnRHRII is also expressed in peripheral places in the body, including lymphocytes, breast, ovary, and prostate. There are three main steps of the gonadotropin-releasing process: receptor binding, mobilization of extracellular calcium and phosphatidylinositol, and granule exocytosis. Continuous exposure of the cells to GnRH in vitro in cell culture resulted in desensitization of the gonadotropic responsiveness [178]. Gonadotropins themselves, gonadal steroids, and inhibin (produced by granulosa cells) modulate the gonadotropin responses to GnRH. The ability of gonadotropes to respond to GnRH depends on the density of GnRHR. Marked differences were observed in the receptor number throughout the estrous cycle: maximal in late diestrus II and proestrus, just prior to ovulation and, likely, it is important for the preovulatory LH to surge and then fall to a low point at estrus [179]. Immunoneutralization, given specific antisera to GnRH on the first day of diestrus, prevented follicular development and E secretion, while administration during the critical period of proestrus afternoon prevented the LH surge and ovulation [180].

## 8. Origin and Development of GnRH Neurons

In the case of hypogonadotropic hypogonadism associated with anosmia, the cause of the disturbance of GnRH functions was previously unknown. The discovery of the origin of the neuroendocrine GnRH neurons was an amazing step forward. It is a very unique phenomenon in the CNS. These neurons develop from the olfactory placode, as it was demonstrated by Schwanzel-Fukuda and Pfaff in 1989 [181]. Immunohistochemistry revealed that GnRH neurons are present in the terminal nerve (nervus terminalis). This nerve arises from the accessory olfactory bulb, enters the nasal cavity through the cribriform plate, and terminates in the vomeronasal organ (VNO). Its sensory endings detect pheromones. With the use of immunohistochemistry, it was explored that in fetal mice, the GnRH neurons derive from the medial olfactory placode and migrate across the nasal septum along the terminal nerve into the brain and reach the septo–preoptic–anterior hypothalamic region. Later, this migration of GnRH neurons was shown in rats as well. GnRH neurons occupy the preoptic area by Ed 16.5. The final destination of migration from the nasal placode to the brain is different in different species. In guinea pigs and primates, the GnRH cells reach the tuberal region; however, in rats, they only reach the anterior hypothalamus [169]. The combination of tract tracers (injected into the ME, intravenously or intraperitoneally) and immunohistochemistry allows the possibility to demonstrate which GnRH neurons reach the capillary loops of the ME, i.e., which neurons are hypophysiotropic [170,182]. Non-hypophysiotropic neurons play a role in the regulation of other functions, such as sexual behavior [183]. The significance of the olfactory placode in the development of GnRH neurons was demonstrated by Saitoh and co-workers [184]. MBH of female rhesus monkeys was lesioned. These animals became anovulatory. Then, a fetal olfactory placode was implanted into the infundibular recess of the third ventricle. Two months later, the implantation of the olfactory placode resulted in resumption of the ovulatory cycle in a high percentage of the animals.

The close developmental relation between the GnRH neurons and the terminal nerve suggests that there may also be a functional link between the two systems. As it was mentioned above, the terminal nerve detects pheromones. The pheromones are bioactive molecules that activate sensory neurons of VNO and modify social and sexual behavior [185,186]. A naive animal responds behaviorally to the presence of pheromones, without any prior experience or exposure, for example: a pup recognizes the source of milk, a male detects the other male and fights with him, and in some species, the estrus cycles can be synchronized if beside the cage of females, another cage is placed containing males. In spontaneously ovulating species (rodents and primates), which are the main topic of this paper, the pheromones do not participate in the main stream of the molecular chain, which elaborates the ovulatory process. However, the pheromones influence the sexual and social behavior. It is an interesting similarity between the GnRH and VNO neurons that pheromones in the sensory neurons of the VNO activate the phospholipase C pathway, as do the GnRH in the target cells [187]. 

## 9. Pulsatility of GnRH

The hypophysiotropic GnRH neurons have a special appearance. Herde and co-workers [188] described the morphology of GnRH neurons using a very elegant set of experiments. They demonstrated that the GnRH neurons are bipolar, with two long dendrite-like processes, one of which projects to the ME. The processes exhibit spines, indicating that these processes are not axons. In the ME, the processes ramify, and the terminals show close contact with the capillary loops. The GnRH processes do not express classical axonal or dendritic markers. These processes were named dendron. Immunohistochemical evidence shows that there are synaptic terminals along the projections, which mediate afferent inputs. Previously, this situation was shown by electron microscopy [189]. A special segment shows a typical action potential initiation site. Researchers suggested that this unusual projection functions both as an axon to conduct action potentials and as a dendrite receiving synaptic input.

It became evident that the pulsatile discharge of GnRH is crucial for the normal release of gonadotropins during ovulation. GnRH neurons secrete their hormones in rhythmic pulses. This property is an intrinsic ability of the GnRH cells, as it was demonstrated using the GT1 (immortalized mature mouse hypothalamic GnRH) neuronal cell line. In a super-fusion system, these cells released GnRH at about four pulses per hour [190,191]. The question arises as to what is the anatomical basis of the fact that the GnRH cells in vivo throw out their products at the same time. Over the years, it has also been hypothesized that GnRH–GnRH synapses (axodendritic and dendrodendritic) or gap junctional dendrodendritic connections synchronize the activity of the cells [192]. It is also an interesting observation that the dendritic spine density of GnRH neurons changes during the estrous cycle. In this mechanism, the gonadal steroids play an important role, i.e., they can modify the spine density of GnRH cells. Chan and co-workers [193] examined the spine density in ovariectomized female mice and compared the results with those of diestrus females. The somatic spine density decreased with the lack of E, while the dendritic spine density did not alter. E2 treatment of ovariectomized mice activated cFos in a subset of GnRH neurons by mimicking the pro-estrous GnRH surge. These activated neurons exhibited a dramatic increase in total spine density. In pubertal female rhesus macaques, quantitative morphometrics demonstrated that the GnRH neurons were opposed by glial processes. Such ensheathment was described earlier in ovariectomized adult animals and was found to be reversible by administration of gonadal steroids [194].

KP, NKB, and Dy expressing neurons (KNDy) are thought to play an important role in the E feedback mechanism, and these neurons are responsible for GnRH-LH pulsatility and preovulatory surge in rodents. These neurons are located in the arcuate and anterior-periventricular regions. These neurons were recently shown to also co-localize with galanin and establish axosomatic and axodendritic connections with GnRH neurons [195]. 

In the last few decades, the afferent connections of GnRH neurons have been explored. Afferent connections are poor in the preoptic area, and more abundant in the hypothalamus. Two groups of afferent connections are identified: stimulating and inhibiting. Glutamate, galanin, neuropeptide-Y, GnRH itself, histamine, norepinephrine, vasoactive intestinal peptide (VIP), substance P, neurotensin, and nitric oxide are known as stimulating factors. GABA, pituitary-adenylate-cyclase-activating polypeptide (PACAP), arginine vasopressin (AVP), cholecystokinin, corticotropin-releasing hormone (CRH), endogen opioids, and gastrin were shown to be inhibiting under tested conditions [195,196,197,198,199,200,201].

## 10. Feedback Mechanism and Pulse and Surge Generators

In the second half of the last century, it was considered that not only is the function of peripheral endocrine glands commanded by the CNS via adenohypophysis, but at the same time, the actual blood level of hormones, which are secreted and released into the general circulation by the target glands, might influence the neural structures primarily involved in the control mechanism. This mechanism was named feedback. E, namely E2, exhibits positive and negative feedback control. The positive E feedback is mediated by KP neurons located in the anterior periventricular area of the hypothalamus [202,203,204]. KP neurons influence the GnRH neurons via the G-protein-coupled receptor, GPR54 [205]. 

It was proposed [203], and nowadays it is generally accepted, that the rostral population of KP neurons form the GnRH surge (surge generator), whereas the arcuate population contributes to a steroid-negative feedback mechanism (pulse generator) [206]. Here, the KP neurons also synthesized NKB and Dy. These neurons form the above-mentioned second group of KNDy neurons [207,208]. We cannot ignore that other factors can influence the course of events. GnRH neurons integrate impulses coming through afferent synapses and intrinsic changes to increase firing rates during the preovulatory GnRH surge [209]. 

## 11. Discovery of the Gonadotropin-Inhibiting Hormone

GnRH has long been considered unusual among hypothalamic neuropeptides in that it appeared to have no hypothalamic antagonist. GnIH was isolated in 2000 in birds and later in mammals. This peptide is a dodecapeptide composed of twelve amino acids. It acts on the pituitary and GnRH neurons through a G-protein-coupled receptor (GPR147) [210]. In rodents, GnIH cell bodies are located in the hypothalamic dorsomedial nucleus and project to other hypothalamic and limbic structures, but not to the ME. Instead, in mammals, GnIH neurons establish synaptic context with neuropeptide-Y (NPY), pro-opiomelanocortin-, orexin-, and melanin-concentrating cells. This morphological observation suggests a role for GnIH in communicating the current energetic status to the reproductive system. Based on the conflicting results, the role of this peptide in the mammalian reproductive functions is not fully understood [211]. 

## 12. The Sequence of Hormonal Events during Ovulation

**Figure 12:**
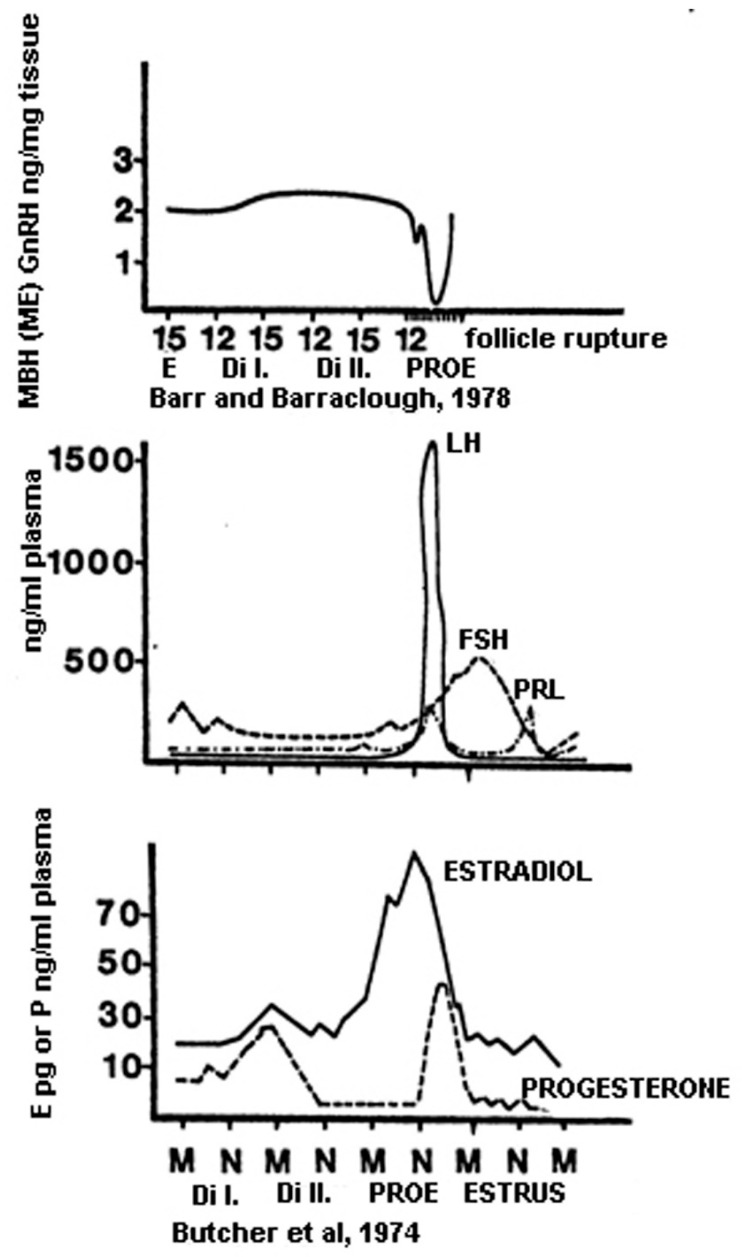
The chart shows the hormone levels during the rat estrous cycle. Abbreviations: E, estradiol; MBH, medial basal hypothalamus; ME, median eminence; E, estrous; DiI, diestrus I; DiII, diestrus II; PROE, proestrus. The chart was constructed on the basis of data of Barr and Barraclough. Temporal changes in medial basal hypothalamic LH-RH correlated with plasma LH during the rat estrous cycle and following electrochemical stimulation of the medial preoptic area in pentobarbital-treated pro-estrous rats. *Brain Research*, Volume 148, Issue 2, 1978, Pages 413–423. 1978 [215]. Elsevier Inc., Amsterdam and Butcher, Collins, and Fugo. Plasma concentration of LH, FSH, PRL, progesterone and estradiol-17 beta throughout the 4-day estrous cycle of the rat. *Endocrinology* 1974, 94, 1704–1708 [216]. ©Oxford University Press.

At birth, the ovaries contain hundreds of thousands of ovarian follicles, each of which contains a primary oocyte, a potential egg cell that is arrested in prophase of the first meiotic division, and granulosa cells. In the next FSH-dependent stage, the surrounding cells differentiate to form theca cells. During childhood, female (and male) reproductive systems are dormant; however, the hypothalamic–pituitary complex is functional. In prepubescent females, gonadotropin levels are low. According to recent evidence, when females hit puberty (in humans, 8–13 years of age; in rats, 30–36 days of age), the hypothalamus begins releasing high levels of GnRH. This phenomenon is preceded by the activity of KNDy neurons located in the pulse generator (arcuate/infundibular nucleus) [212]. Activated KNDy neurons release NKB and Dy to GnRH neurons, initiating the pulsatile secretion of GnRH [213,214]. The pituitary gland responds by secreting increased amounts of gonadotropins, LH and FSH [211]. In females, the granulosa and theca cells in the ovaries respond by secreting E2 and P. When the E2 level reaches a critical level, positive E2 feedback, acting on anteroventral–periventricular KP neurons, generates preovulatory GnRH/LH surge and ovulation. This group of anteroventral–periventricular KP neurons (the surge generator) is responsible for triggering menarche and the onset of menstrual cycling in humans, and the opening of the vaginal membrane and starting the estrus cyclicity in rats. The changes in the GnRH level of the MBH and LH, FSH, PRL, E2, and P in the plasma during the rat estrous cycle are shown in Figure 12. These investigations were carried out by Barr and Barraclough [215] and Butcher and co-workers [216] in the advent of the era when RIA was regularly used in the research work.

During each cycle, a group of follicles is recruited by FSH to reinitiate development [217]. The follicular (proliferative) phase of the cycle prepares the dominant follicle for release during ovulation. When the previous cycle is complete and the corpus luteum stops producing steroids and breaks down, E2, P, and inhibin A levels decrease. Inhibin A is responsible for preventing a primary follicle from being released prematurely by regulating the release of FSH [218]. Positive feedback regulation causes the hypothalamus and the anterior pituitary to release GnRH and FSH into the bloodstream, causing the recruitment of multiple follicles in the ovaries. The increased FSH level allows for aromatase in the granulosa, converting androgen to E, which recruits LH receptors to the granulosa [219]. With these receptors present, the granulosa begins producing P and 17-hydroxyprogesterone, which regulates granulosa cell proliferation and slows follicle growth until it achieves full maturity as a Graafian follicle [220]. This late tertiary follicle attaches to the ovarian wall; concurrently, the E being released by the granulosa causes a gradual thickening of the endometrium lining. The primary oocyte within the Graafian follicle undergoes one meiotic division, from which a secondary oocyte and a polar body are produced. The secondary oocyte proceeds with a second meiotic division, where it is arrested at metaphase of the second division, and the polar body undergoes apoptosis [217]. Inhibin and anti-Mullerian hormone (AMH) play important roles in follicle development and selection. As previously discussed, inhibin A inhibits the release of FSH. As the functioning of ovaries weakens with age, less inhibin A is produced, stimulating a larger release of FSH. As a result, follicles are recruited at increased rates, the overall duration of the follicular period decreases, and immature primary follicles are released for ovulation. Decreasing inhibin A levels, that come with increasing age, can lead to infertility. The secretion of FSH by the hypothalamic pituitary complex also stimulates the secretion of inhibin B by the granulosa cells, which eventually inhibits the secretion of FSH through negative feedback regulation [218]. AMH plays an important role in folliculogenesis. There are multiple theories of how the dominant follicle is selected for ovulation, but the exact mechanism is unknown. One theory is that AMH is involved in the selection, and another is that a follicle expressed the highest amount of FSH receptors and this promoted it as the dominant follicle [220].

The increasing E levels during the follicular phase signals the anterior pituitary gland to rapidly secrete LH, and right before LH levels peak, E levels drastically fall. The sudden increase of LH in the bloodstream causes the activation of the proteolytic enzymes and prostaglandins, which digest collagen in the follicular wall, causing the Graafian follicle to burst [220]. The secondary oocyte is released, and it enters the abdominal cavity and is propelled through the Fallopian tubes. If not fertilized, the oocyte disintegrates following ovulation, but if fertilization does occur, the oocyte undergoes a secondary meiotic division, forming a mature ovum and another polar body. The fusion of the nuclei of the sperm and ovum produces a zygote, which continues through the Fallopian tubes into the uterus, where it implants into the endometrium [217].

Recently, the role of KNDy neurons, using toxin-induced ablation of arcuate KP neurons, was reinvestigated. Selective ablation of KNDy neurons was introduced by the injection of the NKB receptor agonist. Four weeks later, in treated, ovariectomized, E2-primed rats, the LH surge was three times higher than in control rats. In ovary-bearing treated rats, hypogonadism developed, indicating that intact KNDy neurons play a mandatory role in the normal ovarian cycle of rats [221]. ERα disruption in anteroventral–periventricular KP neurons did not delete the negative feedback mechanism but blunted LH surges, and KP neurons exhibited decreased excitability. ERα knocked down in arcuate KP neurons induced disrupted cyclicity [222]. 

In mammals, the exact timing of ovulation seems to depend on the periodicity of external illumination. This was studied through timed lighting of the animal house [223]. In rats, exhibiting regular 4-day cycles, when light is on from 6 a.m. until 6 p.m., the GnRH neurons prepare themselves to release their product into the portal system between 1:30 and 2:00 p.m. This time interval is the “critical period”. Just before this period, ovulation can be blocked if a member of the molecular chain is blocked.

Scharrer hypothesized 60 years ago [224] that light impulses influence the neuroendocrine system (“photo-neuroendocrine system”). Later, it became evident [225,226] that non-image-forming retinal ganglion cells may modulate the neuroendocrine system. Retinal glutamatergic ganglion cells, exhibiting melanopsin and PACAP immunoreactivities as well, form the retino-hypothalamic tract [227]. This pathway reaches the AVP and VIP cells located in the suprachiasmatic nucleus. This cell group was named “biological” or “mind’s clock” [228]. AVP fibers terminate on KP neurons, which create the surge generator. It was shown that AVP, in vitro in brain slices containing KP neurons, dose-dependently increased the firing rate of most KP neurons in the presence of E [229].

It has been known for a long time that constant illumination induces permanent estrous in rats [230]. Bittman [231] investigated the circadian function of three cell types, i.e., KP (forming the pulse and surge generators), AVP (located in the suprachiasmatic nucleus), and GnRH cells, which are involved in the surge generator. E was found to permit circadian AVP signaling at preoptic KP neurons rather than dynamically modulate its activity throughout the estrous cycle.

**Figure 13:**
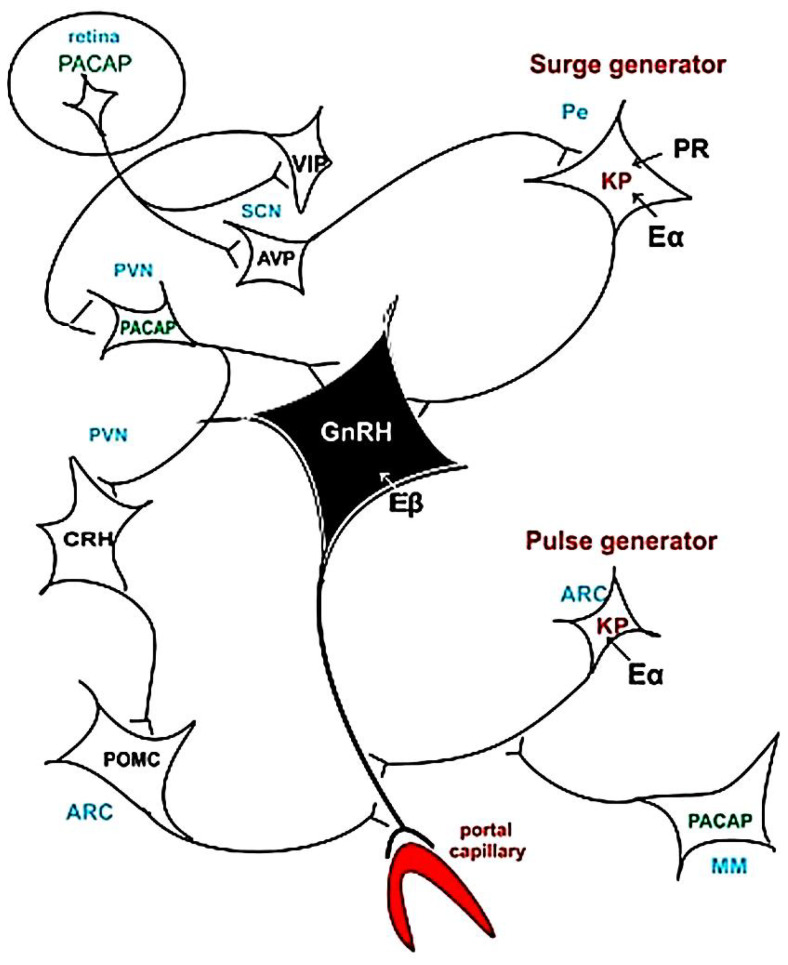
Schematic representation of successive hormonal events during ovulation. Abbreviations: ARC, arcuate nucleus; AVP, arginine vasopressin; CRH, corticotropic-hormone-releasing hormone; Eα, E receptor alpha; Eβ, E receptor beta; GnRH, gonadotropic-hormone-releasing hormone; KP, kisspeptin; MM, medial mammillary nucleus; PACAP, pituitary-adenylate-cyclase-activating polypeptide; Pe, periventricular area; POMC, proopiomelanocortin; PR, progesterone receptor; PVN, paraventricular nucleus; SCN, suprachiasmatic nucleus; VIP, vasoactive intestinal polypeptide. This figure is from Köves, K.; Szabó, E.; Kántor, O.; Heinzlmann, A.; Szabó, F.; Csáki, Á. Current state of understanding of the role of PACAP in the hypothalamo-hypophyseal gonadotropin functions of mammals. *Front. Endocrinol.* 2020. Sec. Reproduction. 11, 88 [199]. ©Elsevier Inc., Amsterdam.

Figure 13 shows a hypothetical pathway of how hormonal events follow each other during the process leading to ovulation.

## 13. Infertility and Assisted Reproduction

Infertility is defined as the failure to achieve pregnancy after 12 months of unprotected sexual intercourse in women <35 years of age. Even as early as in the era of 3500 BC–500 AD, the idea of artificial insemination was known, and involved injecting semen into the reproductive tract of women [232]. However, it was not until the 17th century when physicians really started to think about infertility in a truly scientific manner. The spermatozoa were discovered in 1677 by Leeuwenhoek [233]. About one hundred years later, in 1779, the Italian Spallanzani established that the formation of the embryo is the result of the interaction of the eggs and the sperm [234]. The first successful artificial insemination in humans was conducted by the Scottish John Hunter in the 1780s [235]. 

However, the etiology and pathophysiology of infertility is broad and can be complicated. The scope of this article allows for the exploration of the endocrine and neuroendocrine causes of infertility. Impaired function of the hypothalamic–pituitary–gonadal axis can lead to a wide range of reproductive disorders, including infertility, and is responsible for about 30% of cases of secondary amenorrhea [236]. One of the main controls of reproduction is the tightly coordinated communication between GnRH-containing neurons of the hypothalamus, the gonadotropic cells of the pituitary, and the germ cells of the gonads. Fertility depends on the “GnRH pulse generator”, a neural network located in the infundibular nucleus (equivalent to the arcuate nucleus in rodents), which co-expresses the neuropeptides: KP, NKB, and Dy (KNDy neurons, as it was mentioned above) [203,206]. Inappropriate GnRH secretion results in abnormal LH and FSH secretion, which disrupts folliculogenesis and ovulation. Hypothalamic dysfunction can be caused by structural lesions; however, the most common reason for secondary amenorrhea appears to be functional in nature. Functional dysfunction of the hypothalamus can be a result of a relative energy deficiency in the body, such as that seen with anorexia nervosa, over-exercising, chronic disease, or stress with elevated CRH and cortisol levels, which inhibits GnRH secretion [237,238,239,240,241]. Such imbalances result in disruption of hormones, such as IGF-1, T3/T4, leptin, KP, cortisol, and ghrelin, which disrupt GnRH secretion. In hypothyroidism, a high TRH level induces prolactinemia, which also prevents ovulation [242]. Prolactinomas, PRL-producing tumors of the pituitary gland, result in suppression of GnRH neurons. Dopamine, produced in the arcuate nucleus of the hypothalamus, is known to suppress PRL secretion in the pituitary, via dopamine receptors (D2). Most common treatment modalities of secondary amenorrhea include restoring the negative energy balance, reducing stress, and hormone replacement therapy.

Hormone replacement therapy usually consists of E replacement, and while it corrects amenorrhea, it does not restore GnRH release and does not result in ovulation. In order to restore fertility, treatment should target restoring GnRH pulsatility. This, however, is very difficult, and current therapy involves daily FSH- and LH-containing injections. The development of follicles is tracked via ultrasound, and ovulation is induced by the administration of human chorionic gonadotropin (hCG) when the follicle is over 18 mm in diameter [243].

Clomiphene citrate is a commonly used fertility agent that stimulates FSH secretion. Clomiphene citrate is a racemic mixture of *cis*- and *trans*-isomers. The *cis*-isomer is zuclomiphene, which is estrogenic, and the assigned *trans*-isomer, called enclomiphene, is relatively more antiestrogenic. The racemic mixture used for induction of ovulation is generally composed of 38% zuclomiphene and 62% enclomiphene. Clomiphene citrate is a selective E receptor modulator that has been approved by the Federal Drug Administration (FDA) to treat anovulatory infertility by achieving ovulation. The drug displaces endogenous E from the receptors, resulting in negative estrogenic feedback inhibition, thus increasing gonadotropin secretion [244]. 

There are other emerging avenues of treatment. It is known that genetic leptin deficiency results in hypogonadotropic hypogonadism [245]. Leptin levels are also lower in women with decreased fat mass. While GnRH neurons do not express leptin receptors, in two randomized controlled trials, twice daily administration of recombinant leptin in women with secondary amenorrhea resulted in ovulatory menstrual cycles in 38% and 70%, restored LH pulsatility, and increased levels of E2 and IGF-1 [246,247]. It is likely that KP mediates the effect of leptin on GnRH neurons [248]. Subcutaneous bolus injection of KP-54 stimulates gonadotropin secretion in healthy female subjects [249]. Unfortunately, two weeks of twice daily administration of KP resulted in a loss of efficacy, reduced gonadotropin response, or tachyphylaxis [250]. 

## 14. Conclusions

The aim of this review was to collect the most important experimental data that led to the currently accepted theories about the processes involved in the regulation of ovulation. In Table 1, the most important papers containing the milestone data are shown in chronological order.

## Figures and Tables

**Table 1:** The most important cited papers in chronological order.

Milestone	Year of Discovery	Author	Citation
Recognition of the portal system	1742	Lieutaud	[103]
Description of estrous cycle in rats	1922	Long and Evans	[134]
Discovery of estrogen	1923	Allen and Doisy	[74]
Discovery of progesterone	1929	Corner and Allen	[75]
Vascular ring between the pituitary and the hypothalamus	1930	Popa and Fielding	[19]
Effect of illumination on ovulation	1934	Marshall and Bowden	[18]
Importance of vaginal smears	1941	Papanicolau and Traut	[93]
Neural control of the pituitary gland	1950	Harris	[21]
Description of the magnocellular system	1951	Bargmann and Scharrer	[61]
Restitution of a pituitary graft in the median eminence	1958	Nikitovich-Winer and Everett	[125]
Radioimmunoassay	1959	Yallow and Berson	[94]
Reverse blood flow from pituitary to hypothalamus	1962	Török	[42]
Tuberoinfundibular parvicellular system	1962	Szentágotai	[22]
Description of hypophysiotropic area	1965	Halász and Papp	[124]
Discovery of estrogen receptor α	1967	Jensen	[77]
Chemical characterization of lactogenic hormone	1969	Li	[118]
Description of the structure of the median eminence	1970	Knigge and Scott	[66]
Chemical description of glycoprotein hormones	1971	Pierce	[120]
Isolation and properties of LH- and FSH-releasing hormones	1971	Schally	[145]
Radioimmunoassay of LHRH	1973	Arimura	[151]
Distribution of LHRH in the hypothalamus	1974	King	[156]
Pulsatility of GnRH in the portal blood	1976	Carmel	[165]
Characterization of GnRH receptor	1981	Clayton and Catt	[176]
Development of the pituitary gland	1987	Szabó	[60]
Origin of GnRH from the olfactory placode	1989	Schwanzel-Fukuda and Pfaff	[181]
Demonstration of hypophysiotropic GnRH neurons	1989	Merchenthaler	[170]
Discovery of neurosteroids	1990	Baulieu and Robel	[91]
Discovery of estrogen receptor β	1996	Kuiper	[80]
Molecular background of pituitary differentiation	1998	Treier	[104]
Estrogen α and β knockout models	2000	Dupont	[83]
Description of the surge generator	2008	Herbison	[203]
Description of the pulse generator	2009	Navarro	[206]
Description of the GnRH dendron	2013	Herde	[188]

## Abbreviations

ACTH	adrenocorticotropic hormone
AMH	anti-Müllerian hormone
AVP	arginine vasopressin
BBB	blood–brain barrier
BMP4	bone morphogenetic protein
C	corticotropes
CNS	central nervous system
CRH	corticotropic-hormone-releasing hormone
Dy	dynorphin
ELISA	enzyme-linked immunosorbent assay
E	estrogen
E2	17-β estradiol
Erα	estradiol receptor α
Erβ	estradiol receptor β
FGF	fibroblast growth factor protein
FSH G	follicle-stimulating hormone gonadotropes
GH	growth hormone
GnIH	gonadotropin-release-inhibiting hormone
GnRH	gonadotropin-releasing hormone
GnRHR	GnRH receptor
GPR KP	G-protein-coupled receptor kisspeptin
KNDY	neurons expressing all three peptides: KP, NKB and Dy
KO	knockout
ERKO	E2 receptor knockout
LH	luteinizing hormone
LHRH	luteinizing hormone-releasing hormone
LIM	homeobox subfamily
M	melanocyte
MBH	medial basal hypothalamus
ME	median eminence
MSH	melanocyte-stimulating hormone
NKB	neurokinin B
NPY	neuropeptide-Y
P	progesterone
PACAP	pituitary-adenylate-cyclase-activating polypeptide
Pit1	pituitary-specific transcription factor
PR	progesterone receptor
PRL	prolactin
PROP1	gene encoding Pit1
RIA	radioimmunoassay
SCN	suprachiasmatic nucleus
SF-1	steroidogenic factor-1
Shh	sonic hedgehog protein
TRH	thyroid-stimulating hormone-releasing hormone
TSH	thyroid-stimulating hormone
VEGFA	vascular endothelial growth factor A
VIP	vasoactive intestinal polypeptide
VNO	vomeronasal organ
Wnt5	wingless protein
